# Supplementary material for: Pseudomonas Bacteremia in a Tertiary Hospital and Factors Associated with Mortality
Source: Antibiotics (Basel). 2023 Mar 29;12(4):670. doi: 10.3390/antibiotics12040670 (PMC10135004; doi:10.3390/antibiotics12040670)
Supplement: Supplementary file 1 [file antibiotics-12-00670-s001.zip › antibiotics-2276857-supplementary.pdf]

Table S1. Microbiology of *Pseudomonas* strains isolated from patients' blood cultures.

Pathogen	Number of isolates (% for that specific year)								
	**2015**	**2016**	**2017**	**2018**	**2019**	**2020**	**2021**	**2022**	**2015-2022**
P. aeruginosa	40 (88.89)	46 (90.20)	53 (88.33)	45 (90.00)	48 (85.71)	51 (75.00)	39 (86.67)	35 (79.55)	357 (85.20)
P. alcaligenes	0 (0.00)	0 (0.00)	0 (0.00)	0 (0.00)	0 (0.00)	1 (1.47)	1 (2.22)	0 (0.00)	2 (0.48)
P. fluorescens	0 (0.00)	1 (1.96)	1 (1.67)	0 (0.00)	0 (0.00)	1 (1.47)	0 (0.00)	1 (2.27)	4 (0.95)
P. luteola	0 (0.00)	0 (0.00)	0 (0.00)	0 (0.00)	0 (0.00)	0 (0.00)	0 (0.00)	1 (2.27)	1 (0.24)
P. mendocina	0 (0.00)	0 (0.00)	0 (0.00)	0 (0.00)	1 (1.79)	1 (1.47)	0 (0.00)	0 (0.00)	2 (0.48)
P. oleovorans	0 (0.00)	0 (0.00)	0 (0.00)	0 (0.00)	0 (0.00)	1 (1.47)	0 (0.00)	1 (2.27)	2 (0.48)
P. oryzihabitans	0 (0.00)	1 (1.96)	2 (3.33)	4 (8.00)	4 (7.14)	3 (4.41)	2 (4.44)	1 (2.27)	17 (4.06)
P. putida	3 (6.67)	3 (5.88)	3 (5.00)	0 (0.00)	3 (5.36)	8 (11.76)	3 (6.67)	3 (6.81)	26 (6.21)
P. stutzeri	2 (4.44)	0 (0.00)	1 (1.67)	1 (2.00)	0 (0.00)	2 (2.94)	0 (0.00)	2 (4.54)	8 (1.91)
All *Pseudomonas* strains	45 (10.73)	51 (12.17)	60 (14.32)	50 (11.93)	56 (13.37)	68 (16.23)	45 (10.74)	44 (10.50)	419 (100)

Table S2. Microbiology of *Pseudomonas* strains isolated from patients' blood cultures in regards to acquisition.

Pathogen	Community-acquired (%)	Hospital-acquired (%)	p
P. aeruginosa	81 (90)	276 (83.89)	0.1806
P. alcaligenes	0 (0.00)	2 (0.61)	1.0000
P. fluorescens	1 (1.11)	3 (0.91)	1.0000
P. luteola	0 (0.00)	1 (0.30)	1.0000
P. mendocina	1 (1.11)	1 (0.30)	0.3839
P. oleovorans	0 (0.00)	2 (0.61)	1.0000
P. oryzihabitans	3 (3.33)	14 (4.26)	1.0000
P. putida	1 (1.11)	25 (7.60)	0.0240
P. stutzeri	3 (3.33)	5 (1.52)	0.3770
All *Pseudomonas* strains	90 (21.48)	329 (78.52)	NA

NA: not applicable

Table S3. Microbiology of *Pseudomonas* strains isolated from patients' blood cultures in regards to whether the patient was hospitalized in a ward or in the intensive care unit.

Pathogen	Medical wards (%)	Surgical wards (%)	ICU (%)	p*
P. aeruginosa	125 (88.03)	65 (85.53)	167 (83.08)	0.2715
P. alcaligenes	2 (1.41)	0 (0.00)	0 (0.00)	0.4996
P. fluorescens	2 (1.41)	1 (1.32)	1 (0.50)	0.6244
P. luteola	0 (0.00)	0 (0.00)	1 (0.50)	0.4797
P. mendocina	2 (1.41)	0 (0.00)	0 (0.00)	0.4996
P. oleovorans	0 (0.00)	0 (0.00)	2 (1.00)	0.2295
P. oryzihabitans	2 (1.41)	5 (6.58)	10 (4.98)	0.4593
P. putida	5 (3.52)	5 (6.58)	16 (7.96)	0.1623
P. stutzeri	4 (2.82)	5 (6.58)	4 (1.99)	1.000
All *Pseudomonas* strains	142 (33.89)	76 (18.14)	201 (47.97)	NA

* shows statistical comparison among strains in the ICU and the wards (medical and surgical combined)
ICU: intensive care unit; NA: not applicable

Table S4. Antimicrobial resistance of *Pseudomonas aeruginosa* strains isolated from patients' blood cultures in regards to whether bacteremia was community or hospital acquired.

Antibacterial	Community-acquired (%)	Hospital-acquired (%)	p
Amikacin	13 (14.44)	48 (14.59)	1.0000
Aztreonam	15 (16.67)	91 (27.74)	0.0396
Cefepime	15 (16.67)	69 (20.97)	0.4576
Ceftazidime	16 (17.78)	77 (23.40)	0.3164
Colistin	2 (2.22)	6 (1.82)	0.6828
Gentamicin	12 (13.33)	41 (12.46)	0.8582
Meropenem	11 (12.22)	79 (24.01)	0.0198
Piperacillin	17 (18.89)	76 (23.10)	0.4745
Ticarcillin	25 (27.78)	131 (39.82)	0.0372
Tobramycin	13 (14.44)	57 (17.33)	0.6327
Ciprofloxacin	18 (22.78)	58 (19.66)	0.5321
Pefloxacin	19 (26.39)	59 (22.26)	0.5285

Table S5. Antimicrobial resistance of *Pseudomonas aeruginosa* strains isolated from patients' blood cultures in regards to whether the patient was hospitalized in a ward or in the intensive care unit.

Antibacterial	Medical wards (%)	Surgical wards (%)	Wards (combined) (%)	ICU (%)	p*
Amikacin	25 (20.00)	7 (10.77)	32 (16.84)	29 (17.37)	1.0000
Aztreonam	35 (28.23)	7 (10.77)	42 (22.22)	48 (28.74)	0.1794
Cefepime	30 (24.00)	8 (12.31)	38 (20.00)	40 (23.95)	0.3725
Ceftazidime	33 (26.40)	7 (10.77)	40 (21.05)	43 (25.75)	0.3167
Colistin	0 (0.00)	1 (1.54)	1 (0.53)	2 (1.20)	0.6012
Gentamicin	23 (18.40)	5 (7.69)	28 (14.74)	23 (13.77)	0.8798
Meropenem	30 (24.00)	10 (15.38)	40 (21.05)	42 (25.15)	0.3793
Piperacillin	31 (24.80)	9 (13.85)	40 (21.05)	45 (26.95)	0.2138
Ticarcillin	43 (34.40)	15 (23.08)	58 (30.53)	65 (38.92)	0.1180
Tobramycin	25 (20.00)	9 (13.85)	34 (17.89)	35 (20.96)	0.5031
Ciprofloxacin	29 (27.36)	9 (14.75)	38 (22.75)	35 (23.18)	1.0000
Pefloxacin	32 (34.04)	9 (15.79)	41 (27.15)	33 (24.09)	0.5906

* shows statistical comparison among strains in the ICU and the wards (medical and surgical combined)
ICU: intensive care unit

Table S6. Antimicrobial resistance of the different *Pseudomonas* species isolated from patients' blood.

**Antimicrobial resistance (%)**	***Pseudomonas aeruginosa* (n=357)**	***Pseudomonas alcaligenes* (n=2)**	***Pseudomonas fluorescens* (n=4)**	***Pseudomonas luteola* (n=1)**	***Pseudomonas mendocina* (n=2)**	***Pseudomonas oleovorans* (n=2)**	***Pseudomonas oryzihabitans* (n=17)**	***Pseudomonas putida* (n=26)**	***Pseudomonas stutzeri* (n=8)**
Amikacin	17,086834	0,000000	0,000000	0,000000	0,000000	0,000000	0,000000	0,000000	0,000000
Aztreonam	25,280900	50,000000	50,000000	0,000000	0,000000	50,000000	23,529411	26,923077	12,500000
Cefepime	21,848740	0,000000	25,000000	0,000000	0,000000	0,000000	11,764706	11,538462	0,000000
Ceftazidime	23,249300	50,000000	25,000000	0,000000	0,000000	0,000000	17,647058	15,384615	12,500000
Colistin	0,84033614	0,000000	25,000000	100,000000	0,000000	50,000000	0,000000	0,000000	25,000000
Gentamicin	14,285714	0,000000	25,000000	0,000000	0,000000	0,000000	0,000000	3,8461537	0,000000
Meropenem	22,969187	0,000000	0,000000	0,000000	0,000000	0,000000	0,000000	23,076923	25,000000
Piperacillin	23,809525	0,000000	25,000000	0,000000	0,000000	0,000000	11,764706	15,384615	12,500000
Ticarcillin	34,453780	50,000000	75,000000	0,000000	50,000000	0,000000	29,411764	80,769230	25,000000
Tobramycin	19,327730	0,000000	0,000000	0,000000	0,000000	0,000000	0,000000	3,8461537	0,000000
Ciprofloxacin	22,955975	0,000000	25,000000	0,000000	0,000000	50,000000	0,000000	4,347826	0,000000
Pefloxacin	25,694445	0,000000	33,333332	0,000000	0,000000	0,000000	0,000000	9,523809	16,666666
